# A qualitative exploration of community pharmacist views on providing a mental health and well-being intervention for long-term condition patients

**DOI:** 10.1016/j.rcsop.2025.100629

**Published:** 2025-06-25

**Authors:** Patrick Cabasag, Kebede Beyene, Frederick Sundram, Amy Hai Yan Chan, Holly Wilson, Jeff Harrison

**Affiliations:** a School of Pharmacy, Faculty of Medical and Health Sciences, The University of Auckland, Auckland, New Zealand; b Department of Pharmaceutical and Administrative Sciences, University of Health Sciences and Pharmacy in St Louis, St Louis, MO, United States; c Department of Psychological Medicine, Faculty of Medical and Health Sciences, The University of Auckland, Auckland, New Zealand

**Keywords:** Community pharmacy services, Primary care, Depression, Anxiety, Long-term conditions, New Zealand

## Abstract

**Background:**

Subthreshold depression and anxiety are common, affecting up to 24 % of people over their lifetime and are often associated with long-term conditions. Community pharmacists, who often have an established relationship with people who have long-term conditions, are well placed to identify and address subthreshold depression and anxiety and reduce the risk of progression to clinical mental health disorders.

**Methods:**

Semi-structured individual qualitative interviews were conducted with community pharmacists to explore their perspectives on a pharmacy service for long-term condition patients with subthreshold depression and anxiety. Interviews were audio recorded, transcribed in intelligent verbatim and analysed using a General Inductive Approach.

**Results:**

Eleven purposively selected community pharmacists from diverse backgrounds were interviewed. Four main themes were identified, each with several subthemes. These related to existing support mechanisms for delivering long-term condition and mental health services in community pharmacies, pharmacists' perceptions and attitudes toward service delivery, barriers and facilitators to service implementation, and the design and implementation of a service.

**Conclusions:**

This is the first study to explore community pharmacists' perspectives on a pharmacy intervention for long-term condition patients with subthreshold depression and anxiety. Overall, community pharmacists expressed positive attitudes toward delivering an intervention for people with long-term conditions and subthreshold depression and anxiety. Future work would involve taking a co-design approach to developing and evaluating such an intervention.

## Highlights

• Pharmacists saw value in a mental health service for long-term condition patients.
• Business outlook and pharmacists' professional traits were unique factors outlined.
• Pharmacists highlighted design features that would aid implementation and delivery.

## 1 Background

Subthreshold depression (sDep) and anxiety (sAnx) represent conditions in which people experience signs and symptoms that are significant enough to affect their lives but do not meet the threshold for a clinical diagnosis^1^^,^^2^ and are common conditions in the general population. The prevalence of sDep is estimated to be between 10 % to 24 %,^3^, ^4^, ^5^, ^6^, ^7^ and sAnx is estimated to be up to 13.7 %.^8^

sDep and sAnx cause significant suffering and functional impairment,^2^^,^^9^^,^^10^ and these conditions also have a considerable impact on the quality of life of individuals, resulting in increased healthcare utilisation and considerable economic costs.^11^, ^12^, ^13^ Importantly, sDep and sAnx have been associated with progression to clinical depression and anxiety in up to 35 % of cases^14^ and are linked to long-term conditions (LTCs). Studies have shown that sDep and sAnx are associated with and can exacerbate LTCs, such as diabetes and cardiovascular disease,^15^^,^^16^ demonstrating the bi-directional relationship between mental health conditions and long-term physical conditions.

It has been suggested that intervention may be warranted for up to 80 % of those affected by subthreshold conditions.^17^^,^^18^ However, the mental health system is under pressure.^19^ There is a key opportunity for primary care services to potentially meet the general mental health needs of the population with subthreshold needs.^20^ Over 90 % of people with mental health conditions receive care exclusively in a community care setting,^21^ making a primary care approach feasible.

With the increasing demand and burden on GP and specialist services, an alternative approach is for community pharmacists to have a greater role in mental health care. In some countries, pharmacists are still viewed as medication dispensers and retailers.^22^ Although, in countries such as New Zealand (NZ), Australia, the United Kingdom (UK) and the United States (US), the public has a more accepted view of pharmacists as healthcare providers.^23^^,^^24^ Additionally, it has been shown in the literature that pharmacists often have a large number of professional identities,^25^ leading to confusion about their perceived roles in the healthcare team.

Community pharmacists are widely distributed, accessible and the public's high level of trust in pharmacists^26^ puts them in an ideal position to intervene to reduce the gap of the unmet needs of individuals with mental health conditions.^20^^,^^27^^,^^28^ Brief mental health interventions would be ideal for delivery by community pharmacists as they are relatively low-cost, easy to access without need for prior appointment, do not take too much time to deliver and have good evidence of effectiveness (e.g. self-help interventions such as bibliotherapy^29^^,^^30^). Additionally, community pharmacists, who often have regular contact and an established relationship with people who have long-term conditions, are well placed to intervene and deliver brief interventions.

Developing community pharmacist-led interventions around mental health is relatively new and, therefore, requires comprehensive planning. Many studies have described the barriers and facilitators to implementing new services in a community pharmacy setting^31^^,^^32^ and specifically, there have been studies describing these barriers and facilitators for mental health services in community pharmacies.^33^, ^34^, ^35^ However, there is limited research done on community pharmacists' views on mental health service delivery.^36^^,^^37^ Much of the research around mental health services in community pharmacy focus on medication adherence,^38^^,^^39^ screening,^40^ and medication reviews.^41^ Only one feasibility study has been conducted on a community pharmacy service for LTC patients with sDep in the UK.^42^ No study has explored the views of community pharmacists on delivering interventions for LTC patients with sDep and sAnx.

Therefore, to ensure an effective service that is tailored to the NZ setting is designed, input from a range of key stakeholders, including community pharmacists, other health professionals, policymakers, and service users is needed. The aim of this study is to explore the views and attitudes of community pharmacists on providing a brief intervention for LTC patients with sDep and sAnx and the barriers and facilitators for providing such an intervention.

## 2 Methods

This study employed an exploratory qualitative design to explore community pharmacists' views on providing mental health services for LTC patients with sDep and sAnx. One-on-one semi-structured interviews were used to collect data, focusing on key topics related to the research objectives, while allowing flexibility for additional insights to emerge.

### 2.1 Sampling and recruitment

Purposive sampling was used to select community pharmacists with varied backgrounds and experiences, including age, years of practice, role of the pharmacist, practice setting, and type of pharmacy. A pre-screening questionnaire ensured the inclusion of a diverse range of perspectives relevant to the research topic.

Participants were recruited via advertisement/promotion through professional membership organisations and social media channels, including the Pharmacy Council of New Zealand, the Pharmaceutical Society of New Zealand, the Independent Pharmacists' Association of New Zealand, and the New Zealand Community Pharmacy Facebook Group. Snowball sampling was also used, where potential participants could share information about the study with other potential participants who might be interested.

The sample size was guided by the concept of data saturation, defined in this study as the point when no new themes emerged relevant to the research objectives. To ensure thorough saturation, two additional interviews were conducted when data saturation was reached, as recommended by Yang et al.^43^

### 2.2 Data collection

Semi-structured interviews were used to gather data. The interview schedule was developed based on discussions within the research team, the study objectives, the Consolidated Framework for Implementation Research (CFIR),^44^ and prior literature.^36^ The interview schedule was piloted on an academic pharmacist with community experience, and minor adaptations were made to enhance clarity and relevance. After the first five interviews, the guide was refined to include additional topics brought up by participants. The interview schedule is available in Online Supplementary File 1.

To ensure consistency and deep engagement with the data, all interviews were conducted by the lead author (PC). To accommodate participants' preferences, interviews were offered both in-person and via videoconference, and audio recorded. As a token of appreciation, a $50 supermarket voucher was offered to each participant.

### 2.3 Data analysis

PC transcribed all interviews (*n* = 11) in intelligent verbatim and checked all the transcripts for accuracy against the audio recordings. All participants were offered an opportunity to edit their transcripts, with two participants utilising this option.

The General Inductive Approach (GIA) was used to analyse the interview data,^45^ allowing patterns and meanings to emerge from the data. While data analysis was guided by the research objectives, the findings primarily emerge directly from the data itself, reflecting the GIA's deductive-inductive features. This approach allowed new and unexpected findings to also emerge from the data. NVivo software was used to support the analysis process.

Data analysis began with the preparation of the transcripts. PC continuously revisited the transcripts to familiarise themselves with the data to identify information relevant to the study objectives and to understand the themes emerging from the interviews. Initially, a second coder (HW) coded five de-identified transcripts as a quality assurance step and to develop a coding framework. PC and HW discussed the coding framework, and any discrepancies were resolved by a third author (JH). PC coded the remaining transcripts based on the developed framework. The coding framework was continuously refined as new codes emerged as the research progressed. Only data relevant to the study objectives were coded. The codes were categorised into broader groups, which led to the development of subthemes and themes. For example, the codes of ‘lack of suitable space’ and ‘lack of time’ were grouped under the subtheme of ‘Resource Constraints’, which forms part of the theme of ‘Barriers and Facilitators to Implementation’. Themes were continuously refined and revised until the key and most significant themes were identified. To ensure rigour, JH and PC had regular discussions about the results, which were also presented to the rest of the research team. The research team had discussions about the coding framework to consider alternative themes and clarify where codes might be split or merged, and to identify any missing codes. All members of the research team contributed to the interpretation of the results and data analysis. Any discrepancies were resolved by consensus.

### 2.4 Reflexivity statement

The lead researcher is currently a registered pharmacist with community pharmacy experience. This background provided valuable insights into the study, allowing for a deeper exploration of ideas important to pharmacists. However, it also introduced the potential for unintentional bias due to preconceived ideas about community pharmacy. To mitigate this, several strategies were employed: utilising a second coder, involving team members with diverse backgrounds, and conducting interviews with an open mind.

The research team, comprising individuals with varied professional experiences, contributed to the analysis and interpretation of the findings, ensuring a balanced perspective. Regular discussions and consensus-building further reduced the risk of bias. Additionally, the lead researcher maintained reflexivity throughout the research process, reflecting on how their professional background influenced data collection and analysis.

While the lead researcher's experience facilitated rapport with participants and enriched the data collection process, it was essential to remain vigilant about potential biases. The collaborative approach and the use of multiple coders helped ensure the credibility and trustworthiness of the study's findings.

## Ethics approval

Ethics approval was obtained from the Auckland Health Research Ethics Committee (Reference number - AH25235).

Through semi-structured interviews with community pharmacists, we gathered comprehensive insights into their perspectives on providing mental health services for LTC patients with sDep and sAnx. The following section presents the key themes and findings that emerged from our analysis.

## 3 Results

Eleven participants were interviewed for this study. Two participants initially consented to the interview but did not reply thereafter and were thus not included. The interviews lasted approximately 45 minutes to 1 hour and took place between May and August 2023.

Pharmacy and pharmacist characteristics are outlined in Table 1. We have also mapped the anonymised participants to specific variables in Table 2.

**Table 1:** Community pharmacy and pharmacist characteristics of participants.

Characteristics	Number (n = 11)
**Area in New Zealand**	
**North Island (NI)**	7
**South Island (SI)**	4
**Area classification**	
**Urban**	7
**Rural**	4
**Approximate number of scripts per day**	
**0–200**	5
**201–400**	2
**401–600**	2
**>600**	1
**Not known**	1
**Approximate number of LTC patients**	
**0–100**	6
**101–200**	3
**201–300**	2
**Business Model of Pharmacy**	
**Independent**	6
**Franchise**	3
**Corporate**	2
**Age**	
**25–34**	5
**35–44**	3
**45–54**	2
**55–64**	1
**Number of years as a registered pharmacist**	
**0–9**	4
**10–19**	4
**20–29**	2
**30 and over**	1
**Role as a Pharmacist**	
**Owner**	3
**Dispensary manager**	4
**Regular staff pharmacist**	3
**Locum pharmacist**	1

**Table 2:** Mapping of anonymised participants to specific variables.

Participant	Variables
**CP1**	NI, Urban, 25–34 years old, 0–9 years of experience, Independent, Dispensary Manager
**CP2**	NI, Rural, 35–44 years old, 10–19 years of experience, Franchise, Locum
**CP3**	NI, Urban, 25–34 years old, 0–9 years of experience, Franchise, Regular Pharmacist
**CP4**	NI, Rural, 45–54 years old, ≥30 years of experience, Independent, Regular Pharmacist
**CP5**	NI, Rural, 35–44 years old, 10–19 years of experience, Independent, Owner
**CP6**	SI, Urban, 55–64 years old, 20–29 years of experience, Independent, Dispensary Manager
**CP7**	NI, Urban, 35–44 years old, 10–19 years of experience, Independent, Owner
**CP8**	NI, Urban, 25–34 years old, 10–19 years of experience, Franchise, Dispensary Manager
**CP9**	SI, Rural, 45–54 years old, 20–29 years of experience, Independent, Owner
**CP10**	NI, Urban, 25–34 years old, 0–9 years of experience, Corporate, Dispensary Manager
**CP11**	SI, Urban, 25–34 years old, 0–9 years of experience, Corporate, Dispensary Manager

We identified four main themes, each with several subthemes:

- Existing support mechanisms in community pharmacies,
- Perceptions and attitudes of community pharmacists toward service delivery,
- Barriers and facilitators to implementation, and
- Design and implementation of service

**Fig. 1:**
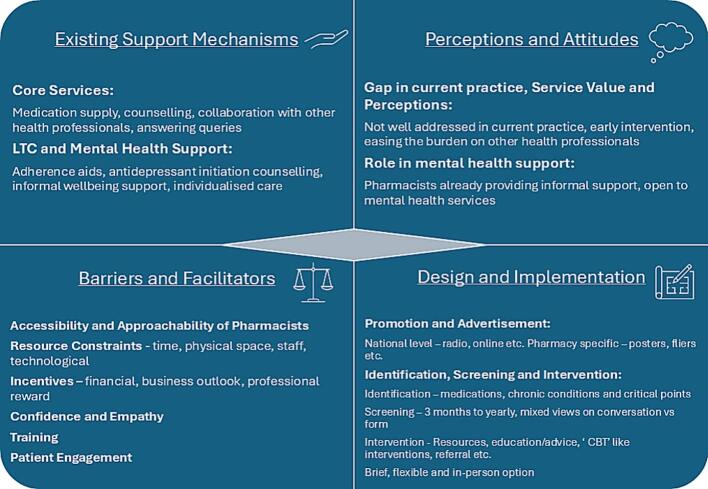
Visual summary of the main themes.

A visual summary of the main themes and subthemes can be found in Fig. 1.

## 4 Theme 1: Existing support mechanisms in community pharmacies

Participants described providing core pharmacy services, such as medication supply, counselling, and collaboration with other health professionals. Support for LTC and mental health patients included adherence aids, informal well-being support, and specific services like antidepressant counselling and administering depot antipsychotic injections.

### 4.1 Core services offered

Participants described providing core pharmacy services to patients, including medication supply and consultation services. Many participants highlighted supports, such as counselling/education, medication supply, and addressing patient concerns or queries. Some participants also mentioned collaborating with other health professionals to discuss medication and non-medication-related issues as a common aspect of their patient care. These are all standard services provided by pharmacists for all patients in the pharmacy.

### 4.2 Support for LTC and mental health patients

Several participants discussed the support offered to both LTC and mental health patients. Adherence aids, such as assisting with medication collection, blister packing, and managing dispensing frequency, were commonly provided to these groups. Few discussed that the primary focus of the LTC services revolved around promoting medication adherence.

In addition to adherence support, participants described providing informal well-being support to both groups of patients.

Some discussed providing counselling for the initiation of antidepressants. One participant noted that in certain regions, pharmacies received funding for counselling patients during the initiation of selective serotonin reuptake inhibitors (SSRIs). Another participant uniquely stated that they administered intramuscular injections (IMIs) for patients on depot antipsychotics.

Participants did not mention any specific formalised support beyond informal check-ins of how their patients were doing for LTC patients with mental health needs. However, some participants emphasised the importance of individualised care and providing support based on the needs of patients.

*“It's more of a holistic approach for each patient.” – CP6*

## 5 Theme 2: Perceptions and attitudes of community pharmacists toward service delivery

Participants highlighted the value of early intervention for LTC patients with sDep and sAnx, noting gaps in current practice and the potential to reduce GP workload. While most felt the service fell within their scope, some expressed concerns about role encroachment and the need for clear communication with GPs.

### 5.1 Gaps in practice, service value, and pharmacist perceptions on the views of other health professionals

Some participants believed that there was a gap in current practice for this type of service as it is not an area well addressed by GPs and not picked up anywhere else.

*“It's probably an area that GPs haven't addressed to any large extent. Suppose they know it's a problem. They may not. I don't know”. – CP9*

Many participants discussed the value of this intervention to prevent people from getting worse.

*“It's very much an ambulance at the bottom of the cliff situation…... I think if you catch things in the early stages, just like anything, it's so much easier to return to a healthy mind and healthy setting than if you've already gone really, really far”. – CP3*

Many participants also highlighted the time constraints faced by other health professionals, such as GPs. As a result, some participants suggested that pharmacists offering certain services could help alleviate the workload of GPs. However, one participant emphasised the need for clear communication with GPs to avoid any potential pushback or perception of role encroachment.

While one participant stated that GPs would be open to the service to reduce their workload, the same participant also expressed doubts about whether other health professionals would trust community pharmacists to deliver this type of service.

*“Perhaps there might even be some professionals out there like counsellors, therapists or psychologists that maybe think that we are not well equipped to handle this kind of thing”. – CP10*

### 5.2 Perceived role in mental health support

Participants indicated that community pharmacists already provide informal support for mental health patients. Some participants mentioned dedicating time to assist patients through conversations and offering well-being support.

One pharmacist expressed a strong motivation to help but admitted uncertainty about how to provide formalised mental health support, suggesting that a structured service could help address this gap.

*“I would love to help these people that I come across every day who look like that they can do with a bit of a pick me up and a bit more help. I don't really know how to go about it”. – CP10*

Most participants felt that the potential service fell within their scope of practice. However, one participant disagreed, noting that with adequate training, pharmacists could still effectively deliver the service.

### 5.3 The association of chronic pain and mental health

Interestingly, some participants specifically mentioned chronic pain and how this condition is closely linked with mental health.

## 6 Theme 3: Barriers and facilitators to implementation

Participants identified several barriers, including lack of suitable space, staff shortages, time constraints, and pharmacists' professional traits. Facilitators included the accessibility and approachability of community pharmacists, existing relationships with LTC patients, training, and the flexibility of community pharmacies.

### 6.1 Perceived accessibility and approachability of community pharmacists

Several participants highlighted that community pharmacists are well-positioned to deliver a potential service for LTC patients with sDep and sAnx. They noted that community pharmacists often serve as the first and frequent point of contact for patients seeking health and medication advice. One participant described pharmacists in a liaison role, acting as a bridge between patients and other healthcare providers.

*“We are in contact with the actual patients and we can liaise with the clinical team as well. So, we're in the middle. So, I think that's a really good point for us to actually intervene if there is a need”. – CP8*

Additionally, many pharmacists stated that they already had existing relationships and rapport with LTC patients.

Participants perceived community pharmacists as readily accessible and available to the public. Many emphasised the flexibility of community pharmacies, noting that patients can visit without an appointment and benefit from extended operating hours.

*“You can just do walk in straight away and access. Some pharmacies open long hours as well. So anytime you need help, you go to a pharmacy straight away”. – CP11*

Contrastingly, some participants mentioned that in rural areas and some areas in the South Island, accessibility was an issue due to patients having difficulties getting to the pharmacy.

Some participants also perceived community pharmacists as approachable in terms of being open and friendly. One participant emphasised that community pharmacies provide a safe and welcoming environment for patients to discuss their concerns. They noted that the casual and informal setting of a pharmacy is often perceived as less intimidating than a GP clinic.

*“Going for a mental health appointment with your GP is a lot more daunting…….. Whereas in a pharmacy, it's a more familiar environment for patients and more casual so may be less confronting for patients….”. – CP3*

### 6.2 Resource constraints

Lack of suitable space in the pharmacy was a barrier brought up by many participants. This was regarding the physical space needed to talk to patients privately. Having consultation spaces and rooms was seen as something needed for this service to support patients' well-being and to maintain a safe place for disclosure.

Many participants stated staff shortages as major barriers in community pharmacies. In particular, some participants who worked in rural pharmacies emphasised the challenges of staff availability and recruitment.

Additionally, some pharmacists suggested that being a sole charge pharmacist could pose a challenge to delivering this type of service. However, one pharmacist disagreed, stating that pharmacists can prioritise and make time to speak with patients when necessary.

*“if someone just discharged from hospital and prescribed heart attack medications, you do need to talk to them for a good 15 minutes about their medications anyway. So, it's the same to me”. – CP11*

Additionally, all participants emphasised that a lack of time as a major barrier in delivering the service. The challenge was linked to multiple factors, including staff availability and the size of the pharmacy, particularly in terms of script load and other services. Some participants also expressed concerns that certain pharmacies might be reluctant to take on additional services, as integrating them into the workflow could be difficult due to time constraints.

To mitigate staff and time-related barriers, some participants suggested involving other staff, such as technicians, interns and occasionally, pharmacy assistants, to help share the workload of the intervention. They emphasised that staff engagement and knowledge of the service could facilitate its successful implementation. Additionally, some participants explained that having sufficient time to plan for the service and having clear communication from the policy makers on what the service entails would help with successful implementation.

One participant was concerned about the fragmentation of care, as community pharmacies do not always have the full picture of everything happening because of technological barriers to accessing clinical information. Many participants suggested that having integrated systems would help pharmacists to deliver the service effectively and efficiently. Additionally, some participants expressed the need to have easy and efficient systems to navigate in terms of clear referral pathways, documentation, and claiming.

Furthermore, participants also discussed having support systems to aid pharmacists in navigating difficult scenarios where they need advice on what to do.

### 6.3 Financial and non-financial incentives

Many participants emphasised that funding is an important factor in encouraging pharmacists to deliver services. They noted that pharmacy is a business, and adequate remuneration is essential for implementing the service. One participant mentioned that funding would also be necessary to provide the resources needed for the service. Additionally, another participant viewed funding as a way to recognise pharmacists as being an important member of the healthcare team. Furthermore, when asked if they would dedicate more time to the service if it were well-funded, all participants agreed they would be more likely to do so.

In addition to funding, non-financial incentives were also seen as motivators for delivering the services. Many participants highlighted the professional reward of seeing their services benefit patients and the personal satisfaction of being able to contribute more as pharmacists.

*“I get a lot out of someone coming back to me and saying hey, you were really helpful that day and that's how I enjoy my work”. – CP4*

Business outlook and aim were also seen as factors in determining whether pharmacies would implement certain services, regardless of the pharmacist's role. A few participants working in independent and franchise pharmacies noted that large commercial chain pharmacies tend to prioritise prescriptions and over-the-counter sales, while smaller and independent pharmacies focus on building close relationships with their patients and providing additional services. One of these participants further suggested that this potential service may not align with the goals of large chain pharmacies.

*“For the more commercial ones where it's more fast paced, it may not because that's not the goal of the pharmacy itself”. – CP1*

Contrary to these statements, one participant from a large chain pharmacy stated that they would be open to providing new services.

*“I know being a corporate pharmacy, we're actually always keen to take on new services. It's not something that we would dismiss for sure”. – CP10*

### 6.4 Confidence and empathy

Most participants expressed confidence in their ability to deliver the service, noting that factors such as mental health training, life experience and the complexity of the service would influence their confidence.

However, while most participants felt confident, some stated a lack of confidence in other pharmacists as a potential barrier. More experienced participants noted that younger and less experienced pharmacists may face greater challenges in delivering such an intervention effectively.

*“I think a lot of pharmacists, especially young ones, is that they don't have the confidence to talk about or to even ask their patients because I think it's more about the unknown like what the patients are going to say”. – CP1*

This was also evident when one participant, who was the only participant with less than 5 years of experience as a pharmacist, expressed doubts about delivering a service because of how patients may react (see more below 6.6 - *Perspectives on patient engagement with pharmacy services*). Additionally, some participants noted that regardless of experience, some pharmacists may not be confident and comfortable delivering the service as it falls outside their expertise and involves a topic often associated with stigma. One participant suggested that regular practice and experience could help pharmacists become more confident in delivering mental health services.

An interesting concept identified in the interviews was the perception that some pharmacists may lack the empathy needed to deliver mental health interventions.

*“I think that some community pharmacists are not as empathetic as they could be”. – CP1*

While a few participants expressed this view, others suggested that perceptions of a lack of empathy could stem from misunderstandings about how some pharmacists present themselves rather than an actual absence of care or compassion.

*“Maybe they didn't mean to show they lacked empathy, but maybe it just came across because it's quite different to how someone else would present themselves. So, maybe it's a misunderstanding perhaps”. – CP10*

Another participant emphasised that empathy and care are essential traits for pharmacists.

*“You would hope that all the pharmacists that made it through pharmacy school would have a bit of empathy and care in them”. – CP10*

Additionally, one participant stated that not all pharmacists may be a ‘people-person’ and prefer to do more technical and less patient-facing tasks. For those lacking confidence or strong interpersonal skills, participants suggested that having a clear guide or protocol for delivering the service could be beneficial.

### 6.5 Training in service delivery

Many participants stated that with the proper training and resources, they would be confident in delivering this type of service. They highlighted that training would be necessary to ensure the service is delivered effectively, prioritises patient safety, and gains credibility.

Participants suggested a range of training courses that may be useful, including simple mental health first aid, handling difficult conversations with patients, health psychology, counselling, and meditation/mindfulness techniques. A few participants emphasised the importance of offering online training for flexibility, with one noting that free training would encourage participation as cost could be a barrier.

Most participants believed that the service should be accreditation-based to ensure pharmacists deliver it appropriately and to a high standard. However, two participants felt that accreditation might not be necessary. One participant argued that pharmacists already possess the basic skills and frequently handle similar tasks in practice, while another saw the service as a natural progression of their current responsibilities.

*“I feel like pharmacists are quite well equipped to deal with these things anyway. It's things we deal with on a daily basis”. – CP10*

### 6.6 Perspectives on patient engagement with pharmacy services

Some participants discussed the stigma surrounding mental health as a significant barrier to patient engagement. Several participants also suggested that generational factors could play a role, with older generations being less aware of or more reluctant to talk about mental health issues. One younger participant expressed concern about approaching patients because of the stigma and how this may scare patients away. A couple of participants stated that due to the stigma of mental health, it would be best not to call the service a ‘mental health’ service but a ‘well-being’ service.

Patient perceptions of the pharmacy as an appropriate setting for such a service were also discussed. One participant stated that pharmacy may not be viewed as sufficiently ‘clinical’ for delivering such a service.

*“whether people think it's too far in the other direction that it's not clinical enough or formal enough of a setting”. – CP3*

Despite this, another participant stated that patient perception is changing on how pharmacy is viewed in terms of delivering clinical services.

*“The last thing on the list is obviously the patient buy-in that they see the pharmacy as the right place to be receiving this service, which I do strongly believe patient perception is changing in that sense”. – CP7*

To overcome these barriers, one participant suggested that informing patients about the rigorous training pharmacists undergo and the scientific validation of the service could build trust and patient engagement. Another participant believed, that over time, patients would be more comfortable and see the service as a regular service provided by pharmacists.

Furthermore, some participants stated that having an established and close relationship with patients would help them engage with the service as they would feel comfortable. While some participants believed a regular pharmacist would be needed to deliver this service as they already have established rapport with the patient, others argued that a lack of familiarity with the pharmacist might also encourage patients to open up. For example, one participant stated that some patients might feel more comfortable sharing their feelings with a health professional they don't know well, as it reduces the fear of embarrassment.

*“Some they want to share with you when they know you more, but some they [would] rather share with professions/health professionals that actually don't know you well then they won't feel shy or feel a bit ashamed when they have to talk about their stress or things like that”. – CP11*

## 7 Theme 4: Design and implementation of service

Participants suggested promoting the service through national and pharmacy-specific advertisements. Patient identification could be based on prescribed medications, medical conditions, and critical life events. Intervention options included providing resources, education, and referrals, with flexibility in the mode of delivery and duration.

### 7.1 Promotion and advertisement

Some participants suggested national-level advertisements of the service targeting the general population through platforms such as radio, online channels, and television. Pharmacy-specific promotional methods were also discussed. This included putting up posters around the pharmacy, advertisements on in-store televisions, and handing out fliers to pharmacy patients.

### 7.2 Patient identification and screening

Many participants proposed identifying individuals based on the medications they are prescribed and the chronic conditions they manage. Others emphasised identifying patients at critical points, such as after a new diagnosis, hospitalisation, or significant personal life events, as well as observing visible changes in mood.

Participants had differing views on the screening process. Some preferred conversational approaches, while others felt that using a form might be effective. Many participants agreed that patient preference would play a crucial role, as some patients might feel more comfortable filling out the screening form at their own convenience.

Participant answers ranged from 3 months to yearly in terms of how often to screen patients. Additionally, one participant mentioned that screening participants at critical points (as mentioned above) and as part of the annual review for the LTC service as potential options.

Participants also had mixed views on how and when to share screening results with patients. Many participants believed telling patients their results immediately, as it capitalises on the opportunity, avoids withholding important information, and ensures timely action on how the patient is currently feeling. In contrast, a few participants suggested delaying the results to allow patients time to process and to show that the pharmacist had thoroughly considered their results.

Additionally, some participants stated that patient preference and circumstances would guide pharmacists when to tell patients their results for the screening.

*“Probably depends on the situation really. Obviously, if someone is in need of urgent help, it requires a more urgent sort of action. Whereas someone that may need a little bit of time just to have a think about things and the best approach to helping someone or getting together some resources that may be useful for someone”. – CP6*

### 7.3 Intervention options

Participants were asked by the interviewer what actions would be appropriate for three groups of patients: high-scoring, low-scoring and subthreshold groups, all defined by the interviewer.

High-scoring groups correspond to those with clinical depression and anxiety or severe mental health issues. For high-scoring groups, all participants agreed that referral to other services would be needed, whether that was their GP, counsellor, or mental health services. One participant stated that it would be good to have the ability for pharmacists to refer to other health professionals.

*“If you go see a doctor, then they can refer people to this and that, but we don't seem to have that ability to do that….. Some more power for that would be good as well”. – CP5*

Low-scoring groups refer to those with minimal to no symptoms of depression and anxiety. Many participants suggested that for low-scoring groups, being able to offer something to patients was needed. These include general counselling/advice and resources for the patients to take away, or leaving the door open for patients to come back if they have any problems in the future. Some participants also stated that re-screening these patients within a reasonable timeframe would be practical, as changes could occur for patients.

Subthreshold groups refer to patients with symptoms of depression and anxiety that are not severe enough for a diagnosis. Participants described a range of management options for patients with sDep and sAnx. Management options, such as giving out resources, providing education and advice, finding the support person for the individual, and referral were the options discussed by participants as potential interventions. Interventions that required training and were slightly more time-intensive were also described and ranged from mindfulness and breathing exercises to ‘CBT like’ interventions such as behavioural activation. Other interventions described included counselling and a motivational interviewing component.

### 7.4 Mode of delivery, intensity, and duration of the intervention

Many participants believed that both the screening and intervention should be brief to fit within pharmacist’ workflow. Suggested durations for the screening ranged from 2 to 10 minutes, while the intervention component was ideally 10 to 30 minutes. Furthermore, participants emphasised that the intervention should be straightforward and clear.

Flexibility was highlighted as a key consideration for the service. Participants discussed the need for both appointment-based and walk-in options to accommodate the varied schedules of pharmacists and patients. Some participants discussed that the complexity of patient lives would make an appointment-based approach unsuitable for everyone. Furthermore, one participant pointed out that busy periods in community pharmacies could require the intervention to be flexible, allowing delivery during quieter times.

*“It really depends on the pharmacy size. Some pharmacies I've worked at, they have enough staff, which they have the luxury to do walk in just like the COVID vaccination….. Sometimes the patient might know the downtime might be 3 or 2, and then they can just make an appointment”. – CP11*

In terms of the mode of delivery, many participants were flexible in how the service was to be delivered, and patient preference and needs were seen as a factor in determining the mode of delivery. Some participants stated that elderly patients may prefer face-to-face as they may not be comfortable with telehealth, while younger patients may be comfortable with digital methods. Some participants discussed that it would also be dependent on the location of the pharmacy, as more rural and remote areas will need telehealth to deliver the service.

*“I think that it needs to be something that is applicable rurally and can be applied by telehealth. In terms of our practice, I don't think we are any more needy than anyone else, but we have complexities being remote rural”. – CP4*

While many participants stated that the service needs to be flexible, some participants believed that in-person was the ideal and preferable method to deliver a more personal and effective service.

*“I think the in-person way is much more personal and that you are going to get more response from the patient, so therefore, that's probably the best way”. – CP1.*

More quotes for the main themes and subthemes can be found in Online Supplementary File 2.

Our findings highlight the existing support mechanisms in community pharmacies, pharmacists' perceptions of service delivery, and the barriers and facilitators to implementing a mental health service for LTC patients with sDep and sAnx. These insights provide a foundation for discussing the implications of our study.

## 8 Discussion

The aims and objectives of the study were to explore the views and attitudes of community pharmacists on a potential service and the barriers and facilitators to implementation. This is the first study to explore the perceptions of community pharmacists relating to the implementation of a service for LTC patients with sDep and sAnx. Many saw the value in an intervention for LTC patients with sDep/sAnx, such as the benefits of early intervention, to meet a gap in services, and to reduce the burden on the healthcare system. Many participants perceived themselves as accessible and approachable health professionals. The accessibility of community pharmacies^26^^,^^46^ and the identification of community pharmacies as a safe place for patients are commonly described in the literature.^47^

Factors related to time, staffing, funding, training, stigma, lack of confidence, and privacy were commonly highlighted by participants in influencing the implementation of a potential service. These are consistent with previous literature and are often perceived as common factors influencing the delivery of any community pharmacy services.^31^, ^32^, ^33^ These factors are not unique to NZ, but it is important to address these barriers upfront. Pragmatic strategies can be employed to help mitigate some of these barriers. For example, appropriate mental health training programs can increase confidence and reduce stigma.^48^ Furthermore, utilising other pharmacy staff can be helpful to ease the burden on pharmacists.^31^

There are currently no nationally funded primary mental health care community pharmacy services in NZ.^49^ Apart from antidepressant counselling, which may be funded in specific regions in NZ, there are no formal mental health services currently delivered by community pharmacists. Interestingly, our findings indicate that community pharmacists seem to regularly intervene for mental health patients informally in practice, primarily through conversations and asking patients how they are doing. This informal support seems to stem from their caring nature and professional duty, rather than from evidence-based practices. While pharmacists have extensive knowledge of depression and anxiety medicines, pharmacists lack formal mental health intervention skills.^50^ A recent study has outlined that community pharmacists in NZ have a high degree of mental health literacy, despite many not having formal training in mental health, but lacked confidence and comfort in providing mental health-related care in comparison to cardiovascular disease.^51^ Formal training in mental health would be the next logical step to upskilling pharmacists to enable them to enhance their mental health-related knowledge and skills and thereby improving their confidence to deliver mental health interventions.

The differing perceptions of pharmacists working in non-large chain and large chain pharmacies on business outlook and aim was an interesting finding. Our findings appear to be consistent with the existing literature. In a NZ study exploring the perceptions of community pharmacists and their role in mental health, the findings suggested that some pharmacies strategically employed more pharmacists to ensure safe practice and focus on patient-centred care. Furthermore, one participant questioned whether corporate pharmacies would have the same model to focus on patient-centred care.^36^ Another NZ study has also shown that doctors were concerned about these ‘discount’ pharmacies compromising patient care.^52^ While the business model of the pharmacy may have an influence on patient care, other factors, such as resource constraints and individual pharmacists, may also have a significant effect on the quality of patient care, highlighting the complex and multifactorial nature of community pharmacy. Despite the lack of evidence of compromised care in large chain pharmacies, there seems to be negative perceptions of large chain pharmacies solely because they are discounters. Further studies are needed to explore the perceptions of the public, pharmacists and other health professionals on discount pharmacies and the factors that influence the quality of pharmacy services.

A surprising finding was that some participants believed that some pharmacists had a lack of empathy, which is an idea not well described in the literature. Literature has shown that a lack of empathy could result from many factors, such as physical and emotional exhaustion from their workload and burnout.^53^ It has been shown in the literature that burnout and stress are common in pharmacists. A NZ study has also indicated that many early career pharmacists experience burnout and stress,^53^ which in turn may be factors for a lack of empathy. These findings are concerning as empathy is a critical trait needed to provide quality care to patients. Furthermore, early career pharmacists are the future of the profession, and if this issue persists, it could influence the quality of patient care and health outcomes. A stronger focus on developing empathy skills at university could be a sensible approach, as this is a crucial period of development for future pharmacists. Additionally, having role-play scenarios as part of the training for the service may also be useful. Research into the public's perceptions of whether community pharmacists are seen as empathetic healthcare professionals and the factors that influence empathetic patient care in community pharmacists would be useful. Once these questions have been answered, pragmatic strategies can be incorporated to address the underlying causes of a lack of empathy.

The perceived appropriateness of the pharmacy setting to deliver clinical services, such as a service for LTC patients with sDep and sAnx, also came up as a discussion point. While there has been an expansion in the role and the services provided by pharmacists, research has shown that patients are either unaware that pharmacists are capable of a role, choose not to use pharmacists or believe the role is not appropriate for pharmacists.^54^ The pharmacists' role is always evolving and may explain why patients are unaware or believe pharmacists are not capable of delivering different services. Advocating and informing the public that pharmacists are ‘versatile’ and ‘holistic’ health professionals may help the public understand that pharmacists can deliver a wide range of services. However, studies have shown promising results in consumer perceptions of viewing pharmacy as an appropriate setting for mental health service delivery.^55^ While this is the case, there seems to be a lack of awareness by the public of how pharmacists can support people with mental health needs. In an Australian study, the general perceptions of the public on the role of pharmacists in mental health care are mostly focused on the traditional roles of medicine distribution, counselling and referral.^56^ As highlighted in our study, there would need to be public education if pharmacists were to deliver a service for LTC patients with sDep and sAnx. There would need to be a shift in the perceptions of pharmacists as pill counters and retailers to clinical health professionals who are able to provide non-pharmacological mental health services. Realistically, this will take time, but by constantly advocating, educating and demonstrating the capabilities of pharmacists to the public, the narrative will eventually change.

The patient-pharmacist relationship was also identified as being critical in mental health care by patients and can affect whether patients view the pharmacy as a safe space.^47^ Furthermore, a higher level of relationship between the two has been shown to influence the willingness of the patient to accept services provided by the pharmacist.^57^ This may potentially be a barrier for locum pharmacists when providing mental health services, as often, they will not have an established relationship with patients. Interestingly, it was discussed that not having an established relationship could also potentially be a facilitator. This will largely depend on patient preferences, but this should not stop locums from being able to provide this service, as some patients may prefer this. It would be plausible for locums to have the option to upskill in this area, if they wish. Much of the literature focuses on the challenges in patient care for locum health professionals and pharmacists.^58^^,^^59^ Further research on patient perceptions of locum pharmacists and any differences in the quality of care between locum and regular pharmacists is needed.

As outlined in the methods section, the CFIR was used primarily to guide the development of the interview schedule. While the GIA was used to analyse the data and generate themes, some of the findings, as would be expected, reflect domains of the CFIR.^44^ The findings of this study largely relate to the inner setting and individuals domain. For example, resource constraints, incentives, training, existing relationships, and patient perceptions of pharmacy can be mapped onto these domains. The CFIR can provide a structure to help systematically assess the barriers and facilitators to implementation. Future research should ensure the perspectives of individuals involved in the outer domain are gathered, as these are essential for creating an effective intervention.

## 9 Limitations and strengths

Participants were likely to be more passionate about the pharmacy profession and therefore, more likely to provide positive answers regarding their thoughts on the service, which may affect study generalisability. Whilst some barriers outlined in this study can be translated into different settings, the nature of community pharmacy and the role of pharmacists are different in other countries, and so, there is a limitation in the generalisability of the findings to other countries. The researchers also have experience as a community pharmacist themselves, so this may have introduced some biases. However, strategies such as using the GIA, multiple coders, and regular discussions of the results with the research team helped reduce this.

Despite the limitations, examining the perspectives of community pharmacists in this novel area is valuable. This the first study to explore the perceptions of community pharmacists' views on a service for LTC patients with sDep and sAnx. The study presents new findings on the attitudes and perceptions of community pharmacists on a service for LTC patients with sDep and sAnx and what that may look like in practice. A key strength of this study is the diverse participant recruitment, enabling us to capture a broad range of perspectives.

## 10 Recommendations

We have formulated some recommendations for future research and the next steps resulting from this study. This can be found in Table 3.

**Table 3:** Recommendations.

▪ **Community pharmacies should be integrated to play a key role in supporting LTC patients with mental health needs in primary care.**
▪ **Formal training in mental health for community pharmacists e.g. mental health first aid, brief interventions.**
▪ **Further studies exploring the perceptions of the public on large chain pharmacies and the factors that influence patient care.**
▪ **Further research into the perceptions of the public on whether community pharmacists are empathetic and the factors that influence empathetic patient care.**
▪ **Continual education and demonstration to the public on the capabilities of pharmacists.**

### 10.1 Implications of findings

This study adds to the existing literature on community pharmacists' views on mental health service delivery and the factors influencing the implementation of mental health service delivery. If a pharmacist-led service were to be successfully implemented in practice, it would have the potential to reduce the number of individuals progressing from subthreshold to clinical depression and anxiety, therefore reducing the overall prevalence of LTC patients with depression and anxiety. Furthermore, this research will also inform future primary care mental health services/interventions by highlighting key elements that need to be considered in the service design process.

This study indicates there is potential for a pharmacist-led service, but participants identified crucial elements that should be addressed in subsequent service design and implementation activities. The service would need to establish prerequisites, including having sufficient space and appropriate training, for community pharmacies to be able to deliver such a service. Moreover, it is important to ensure adequate remuneration is provided to reflect the time commitment for pharmacies to be able to deliver the service. It was also discussed that utilising other pharmacy staff members may help mitigate the time constraints that pharmacists may face. Allowing appropriately trained pharmacy technicians and interns to screen participants would be an efficient strategy to help pharmacists deliver the service. Additionally, the service needs to be brief and have a tightly defined scope for pharmacists to deliver a service. Flexibility for pharmacists and service users, in terms of mode of delivery and walk-in/appointments, is essential to tailor the intervention to the needs of different areas, community pharmacies and patients. Participants highlighted that clear referral pathways are needed to aid pharmacists in delivering the service when things are outside their scope of practice. The ability to communicate and share information with other health professionals would be valuable to deliver this service to ensure other health professionals, such as GPs, are aware of the care their patients are receiving, therefore optimising care, and reducing the need for patients to retell their stories. Ideally, this would be a structured electronic health record. Furthermore, an important concern that was raised was the perception of the public and other health professionals that pharmacists may not be capable and suitable health professionals to deliver this service. It appears that there will be a need for public promotion and awareness of the service to inform the public that pharmacists have received appropriate training and are appropriate health professionals to deliver a service. Lastly, for any services related to mental health, naming the service a ‘well-being’ or ‘wellness’ service would make it more patient-friendly because of the stigma still attached to mental health.

Existing research suggests that co-design can bring benefits to research processes and outcomes, including improving the creative process, reducing development costs and a better understanding of user needs. Therefore, a service of higher quality and acceptability will be more likely to be produced.^60^^,^^61^ Gathering the thoughts of other key groups, such as consumers and other stakeholders, such as other health professionals and policymakers would be the next logical steps. Further research analysing the effectiveness and cost-effectiveness of interventions in this area is needed to help draw conclusions on whether community pharmacists can take on these roles. Due to the limited studies in this area, the design would need to undergo a feasibility/pilot test to ensure that it can be delivered in a community pharmacy setting.

## 11 Conclusion

This is the first study to explore community pharmacist's thoughts on an intervention for LTC patients with sDep and sAnx. Many participants saw the value of such an intervention and described a range of factors that need to be considered when designing an intervention. These findings can be used to inform the future design of an intervention, with the potential to address sDep and sAnx in a community pharmacy setting. Future work would involve taking a co-design approach to developing and evaluating an intervention for LTC patients with sDep and sAnx in a community pharmacy setting.

## Funding sources

This study is supported by the 10.13039/501100001505Health Research Council of New Zealand [HRC 23/181] and the 10.13039/501100001537University of Auckland [9447/PCAB813].

## CRediT authorship contribution statement

**Patrick Cabasag:** Writing – review & editing, Writing – original draft, Visualization, Project administration, Methodology, Funding acquisition, Formal analysis, Data curation, Conceptualization. **Kebede Beyene:** Writing – review & editing, Supervision, Project administration, Methodology, Funding acquisition, Conceptualization. **Frederick Sundram:** Writing – review & editing, Supervision, Project administration, Methodology, Funding acquisition, Conceptualization. **Amy Hai Yan Chan:** Writing – review & editing, Supervision, Project administration, Methodology, Funding acquisition, Conceptualization. **Holly Wilson:** Formal analysis, Data curation. **Jeff Harrison:** Writing – review & editing, Supervision, Project administration, Methodology, Funding acquisition, Conceptualization.

## Declaration of competing interest

The authors declare that they have no known competing financial interests or personal relationships that could have influenced the work reported in this paper.

## Data Availability

The data supporting the findings of the study are available within the article and its supplementary materials.

## Appendix A Supplementary data

Supplementary material 1 Image 1

Supplementary material 2 Image 2

## References

[1] Rodríguez M.R., Nuevo R., Chatterji S., Ayuso-Mateos J.L. (2012). Definitions and factors associated with subthreshold depressive conditions: a systematic review. BMC Psychiatry.

[2] Gilmour H. (2016). Threshold and subthreshold generalized anxiety disorder (GAD) and suicide ideation. Health Rep.

[3] Kessler R.C., Zhao S., Blazer D.G., Swartz M. (1997). Prevalence, correlates, and course of minor depression and major depression in the National Comorbidity Survey. J Affect Disord.

[4] Rucci P., Gherardi S., Tansella M. (2003). Subthreshold psychiatric disorders in primary care: prevalence and associated characteristics. J Affect Disord.

[5] Jongenelis K., Pot A.M., Eisses A.M., Beekman A.T., Kluiter H., Ribbe M.W. (2004). Prevalence and risk indicators of depression in elderly nursing home patients: the AGED study. J Affect Disord.

[6] Zhang R., Peng X., Song X. (2023). The prevalence and risk of developing major depression among individuals with subthreshold depression in the general population. Psychol Med.

[7] Volz H.-P., Stirnweiß J., Kasper S., Möller H.-J., Seifritz E. (2023). Subthreshold depression – concept, operationalisation and epidemiological data. A scoping review. Int J Psychiatry Clin Pract.

[8] Volz H.-P., Saliger J., Kasper S., Möller H.-J., Seifritz E. (2022). Subsyndromal generalised anxiety disorder: operationalisation and epidemiology – a systematic literature survey. Int J Psychiatry Clin Pract.

[9] Backenstrass M., Frank A., Joest K., Hingmann S., Mundt C., Kronmüller K.T. (2006). A comparative study of nonspecific depressive symptoms and minor depression regarding functional impairment and associated characteristics in primary care. Compr Psychiatry.

[10] Mathieson F., Collings S., Dowell A. (2009). Sub-threshold mental health syndromes: finding an alternative to the medication of unhappiness. J Prim Health Care.

[11] Cuijpers P., Koole S.L., van Dijke A., Roca M., Li J., Reynolds C.F. (2014). Psychotherapy for subclinical depression: meta-analysis. Br J Psychiatry.

[12] Cuijpers P., Smit F. (2004). Subthreshold depression as a risk indicator for major depressive disorder: a systematic review of prospective studies. Acta Psychiatr Scand.

[13] Haller H., Cramer H., Lauche R., Gass F., Dobos G.J. (2014). The prevalence and burden of subthreshold generalized anxiety disorder: a systematic review. BMC Psychiatry.

[14] Schreuder M.J., Wigman J.T.W., Groen R.N., Wichers M., Hartman C.A. (2021). On the transience or stability of subthreshold psychopathology. Sci Rep.

[15] Meeks T.W., Vahia I.V., Lavretsky H., Kulkarni G., Jeste D.V. (2011). A tune in “a minor” can “b major”: a review of epidemiology, illness course, and public health implications of subthreshold depression in older adults. J Affect Disord.

[16] Grenier S., Richer M.-J., Byrne G.J., Pachana N.A. (2021). Anxiety in Older People: Clinical and Research Perspectives.

[17] Wagner H.R., Burns B.J., Broadhead W.E., Yarnall K.S., Sigmon A., Gaynes B.N. (2000). Minor depression in family practice: functional morbidity, co-morbidity, service utilization and outcomes. Psychol Med.

[18] McManus S., Meltzer H., Brugha T., Bebbington P.E., Jenkins R. (2009). Adult psychiatric morbidity in England: results of a household survey.

[19] Patterson R., Durie M., Disley B. (2018).

[20] Anthony Dowell S.G., Collings Sunny (2009).

[21] Ministry of Health (2017).

[22] Majchrowska A., Bogusz R., Nowakowska L., Pawlikowski J., Piątkowski W., Wiechetek M. (2019). Public perception of the range of roles played by professional pharmacists. Int J Environ Res Public Health.

[23] Eades C.E., Ferguson J.S., O’Carroll R.E. (2011). Public health in community pharmacy: A systematic review of pharmacist and consumer views. BMC Public Health.

[24] Ekenga V., Bailey-Wheeler J., Sarpong D., Hart T., Earls M. (2018). Patients’ perception of community pharmacists as healthcare providers and willingness to participate in pharmacist services: a pilot study. J Pharm Health Serv Res.

[25] Elvey R., Hassell K., Hall J. (2013). Who do you think you are? Pharmacists’ perceptions of their professional identity. Int J Pharm Pract.

[26] Confederation N. (2013).

[27] Dowell A.G.S., Collings (2007).

[28] Dew K, Dowell A, McLeod D, Collings S, Bushnell J. "this glorious twilight zone of uncertainty": mental health consultations in general practice in New Zealand. Soc Sci Med (1982). 2005;61(6):1189–200. 10.1016/j.socscimed.2005.01.025 15970230

[29] Jorm A.F., Griffiths K.M. (2006). Population promotion of informal self-help strategies for early intervention against depression and anxiety. Psychol Med.

[30] Gellatly J., Bower P., Hennessy S., Richards D., Gilbody S., Lovell K. (2007). What makes self-help interventions effective in the management of depressive symptoms?. Meta-Anal. Meta-Regress. Psychol. Med..

[31] Weir N.M., Newham R., Dunlop E., Bennie M. (2019). Factors influencing national implementation of innovations within community pharmacy: a systematic review applying the consolidated framework for implementation research. Implement Sci.

[32] Shoemaker S.J., Curran G.M., Swan H., Teeter B.S., Thomas J. (2017). Application of the consolidated framework for implementation research to community pharmacy: A framework for implementation research on pharmacy services. Res Social Adm Pharm.

[33] Crespo-Gonzalez C., Dineen-Griffin S., Rae J., Hill R.A. (2022). A qualitative exploration of mental health services provided in community pharmacies. PloS One.

[34] Murphy A.L., Phelan H., Haslam S., Martin-Misener R., Kutcher S.P., Gardner D.M. (2016). Community pharmacists’ experiences in mental illness and addictions care: a qualitative study. Subst Abuse Treat Prev Policy.

[35] Calogero S., Caley C.F. (2017). Supporting patients with mental illness: deconstructing barriers to community pharmacist access. J Am Pharm Assoc.

[36] Morris C., Wong M., McKinlay E. (2021). A qualitative study of community pharmacists’ perceptions of their role in primary mental health care in New Zealand. Int J Pharm Pract.

[37] Crump K., Boo G., Liew F.S. (2011). New Zealand community pharmacists’ views of their roles in meeting medicine-related needs for people with mental illness. Res Social Adm Pharm.

[38] Al-Jumah K.A., Qureshi N.A. (2012). Impact of pharmacist interventions on patients’ adherence to antidepressants and patient-reported outcomes: a systematic review. Patient Prefer Adherence.

[39] Rubio-Valera M., Serrano-Blanco A., Magdalena-Belío J. (2011). Effectiveness of pharmacist Care in the Improvement of adherence to antidepressants: A systematic review and Meta-analysis. Ann Pharmacother.

[40] Miller P., Newby D., Walkom E., Schneider J., Li S.C. (2020). Depression screening in adults by pharmacists in the community: a systematic review. Int J Pharm Pract.

[41] Gisev N., Bell J.S., O’Reilly C.L., Rosen A., Chen T.F. (2010). An expert panel assessment of comprehensive medication reviews for clients of community mental health teams. Soc Psychiatry Psychiatr Epidemiol.

[42] Chew-Graham C.A., Kitchen C.E.W., Gascoyne S. (2022). The feasibility and acceptability of a brief psychological intervention for adults with long-term health conditions and subthreshold depression delivered via community pharmacies: a mixed methods evaluation—the community pharmacies mood intervention study (CHEMIST). Pilot Feasibility Stud.

[43] Yang L., QI L, ZHANG B. (2022). Concepts and evaluation of saturation in qualitative research. Advances. Psychol Sci.

[44] Damschroder L.J., Reardon C.M., Widerquist M.A.O., Lowery J. (2022). The updated consolidated framework for implementation research based on user feedback. Implement Sci.

[45] Thomas D.R. (2006). A general inductive approach for analyzing qualitative evaluation data. Am J Eval.

[46] Valliant S.N., Burbage S.C., Pathak S., Urick B.Y. (2022). Pharmacists as accessible health care providers: quantifying the opportunity. J Manag Care Spec Pharm.

[47] Mey A., Knox K., Kelly F. (2013). Trust and safe spaces: mental health consumers’ and Carers’ relationships with community pharmacy staff. Patient - Patient-Centered Outcomes Res.

[48] Crespo-Gonzalez C., Dineen-Griffin S., Rae J., Hill R.A. (2022). Mental health training programs for community pharmacists, pharmacy staff and students: A systematic review. Res Social Adm Pharm.

[49] McDonald J., Morris C., Pledger M. (2021). A national survey of pharmacists and interns in Aotearoa New Zealand: provision and views of extended services in community pharmacies. BMC Health Serv Res.

[50] Kirschbaum M., Peterson G., Bridgman H. (2016). Mental health first aid training needs of Australian community pharmacists. CurrPharm Teaching Learning.

[51] Rimal R., Lin J., Yan Chan A.H., Chen T.F., Sheridan J., Sundram F. (2022). A national study of the mental health literacy of community pharmacists. Res Social Adm Pharm.

[52] Addison C., Taylor D. (2023). The pharmacist as safety net: an interview-based study of the intersecting dependencies between doctors and pharmacists. J Pharm Policy Practice.

[53] Hobeika E., Hallit S., Sacre H., Obeid S., Hajj A., Salameh P. (2020). Factors associated with empathy among community pharmacists in Lebanon. J Pharm Policy Practice.

[54] Schommer J.C., Gaither C.A. (2014). A segmentation analysis for pharmacists’ and patients’ views of pharmacists’ roles. Res Social Adm Pharm.

[55] Hall B., Kelly F., Wheeler A.J., McMillan S.S. (2021). Consumer perceptions of community pharmacy-based promotion of mental health and well-being. Health Promot J Austr.

[56] Singleton J., Stevens J.E., Truong R. (2023). Consumer knowledge of mental health conditions, awareness of mental health support services, and perception of community pharmacists’ role in mental health promotion. Int J Pharm Pract.

[57] Adekunle O.A., Olson A.W., Schommer J.C., Brown L.M. (2023). Influence of patient-pharmacist relationship on willingness to accept pharmacist-provided services. J Am Pharm Assoc.

[58] Ferguson J., Stringer G., Walshe K. (2024). Locum doctor working and quality and safety: a qualitative study in English primary and secondary care. BMJ Quality & Safety.

[59] Shann P., Hassell K. (2006). Flexible working: understanding the locum pharmacist in Great Britain. Res Social Adm Pharm.

[60] Steen M., Manschot M., Koning Nd (2011). Benefits of co-design in service design projects. Int J Des.

[61] Slattery P., Saeri A.K., Bragge P. (2020). Research co-design in health: a rapid overview of reviews. Health Res Policy Syst.

