# Supplementary material for: A qualitative exploration of community pharmacist views on providing a mental health and well-being intervention for long-term condition patients
Source: Explor Res Clin Soc Pharm. 2025 Jun 25;19:100629. doi: 10.1016/j.rcsop.2025.100629 (PMC12270050; doi:10.1016/j.rcsop.2025.100629)
Supplement: Supplementary file 1 — Supplementary material 1 [file mmc1.docx]

**Online Supplementary File 1: Interview Schedule**

**Questions for Pharmacists**

**Current Practice**

- What are the ways you support/intervene for LTC patients?
- What about people with mental health needs?
- What are your thoughts on pharmacists doing more in the mental health area?

**Initial thoughts on service**

- Do you think there is a role pharmacists can play in delivering a service for chronic condition patients with subthreshold depression and anxiety? Please explain your answer.
- How useful do you think this would be in your setting?

**Confidence and comfortability in delivering service**

- How confident and comfortable do you think you and your colleagues would be delivering this service to patients with chronic conditions?
- What would make you and your colleagues more confident and comfortable?
  - Prompts – training, support, resources etc.

**Motivation delivering service**

- What would motivate you to deliver this service/What would make you want to deliver a service like this?

**Needs to deliver service**

- What do you think are the barriers and facilitators to providing this new service?
- What would you need to be able to deliver this service?
  - Prompts - funding, space, equipment/materials, training/support, staffing etc.

**Intervention in practice**

- How do you and your pharmacy cope whenever a new service is implemented? Prompts - strategies, techniques, methods, protocols, procedures, support, training etc.
- What do you find difficult whenever implementing a new service?
- For the service I have proposed, what problems do you see in trying to implement this into everyday practice/considerations needed to implement this?

**GP and other health professionals**

- What other health professionals might need to be involved in this service?
- What are the advantages and disadvantages of having pharmacists deliver this service over a GP?

**Ideal service – From start to finish**

- We’ll walk through the whole thing in order of what the service could look like theoretically and stop at different points
- Identification
  - How would you go about identifying patients with chronic conditions and letting them know about this service, practically?
- Screening
  - Do you think this is something pharmacists can practically do? Please explain your answer.
  - How would you screen the patients? E.g. phone, in-person or online? Talk or form? Prompts – what about those that don’t come in for scripts and get them on behalf of someone?
  - How can we encourage patients to be screened and use the service?
  - What would be practical in terms of the time to screen patients and how often would you screen them?
  - How would you approach patients in telling them the results?
- Intervention
  - What do you think might be feasible in terms of intervention components in a pharmacy setting? Prompts – screening, self-help, talk therapy, signposting etc.
  - After screening, you’ll end up with roughly 3 groups of people - those with no to little symptoms, those with concern but not diagnosed and those with concerning results and require further assessment for depression and anxiety. What do we do with each group? Prompts - How would referral work?
  - How would you get patients to come in for this service? Prompts – appointments or walk-ins?
  - How would you deliver the intervention – mode of delivery? Prompts - In-person, phone, workbook or online
  - What would be feasible in terms of time pharmacists take for the intervention?
  - How often do you think we should follow up with patients after initial intervention?
  - What other intervention components do you think might be feasible on what pharmacists can do for these patients in a pharmacy setting?

**Last questions and thoughts**

- To finish off, what are the 3 most important characteristics/elements that are essential to you that this service ‘must’ have?
- Discuss interesting/key ideas other pharmacists have said and what they think of that.
- Any other thoughts, suggestions, comments and issues to add?
