# Supplementary material for: A qualitative exploration of community pharmacist views on providing a mental health and well-being intervention for long-term condition patients
Source: Explor Res Clin Soc Pharm. 2025 Jun 25;19:100629. doi: 10.1016/j.rcsop.2025.100629 (PMC12270050; doi:10.1016/j.rcsop.2025.100629)
Supplement: Supplementary file 2 — Supplementary material 2 [file mmc2.docx]

**Online Supplementary File 2 – More Quotes for the Main Themes and Subthemes**

Please refer to Table 2 in the main manuscript for the mapping of anonymised participants to variables.

**More quotes for Theme 1: Existing support mechanisms in community pharmacies**

| Subthemes | Quotes |
| --- | --- |
| **Core services offered**  **Support for LTC and mental health patients** | *“There's the standard pharmacist stuff like medicine provision [and] advice around medicines”. – CP2*  *“To counsel and support and give advice”. – CP9*  *“We will just address their concerns”. – CP9*  *“We would liaise with their GP or any specialists that are involved. We do lots of interacting between different prescribers”. – CP7*  *“The long-term condition service is mainly around adherence. So, it's supporting them to adhere to the medication regimen”. – CP4*  *“We administer IMIs in store as well. So, if someone's on an antipsychotic IMI, then we can administer that”. – CP7*  *“We would probably start by just looking at cutting down someone's dispensing……. If three months was maybe a little bit overwhelming for a person, we would start by looking at reducing that”. – CP7*  *“the midCPG used to fund pharmacists for a full on consult if anyone was starting a new SSRI”. – CP3*  *“Asking people how they are”. – CP2* |

**More Quotes for Theme 2: Perceptions and attitudes of community pharmacists toward service delivery**

| Subthemes | Quotes |
| --- | --- |
| **Gaps in practice, service value, and pharmacist perceptions on the views of other health professionals**  **Perceived role in mental health support**  **The association of chronic pain and mental health** | *“I think there's 100% a demand out there and I think it's probably a patient group that really slip through and don't get picked up”. – CP7*  *“the message needs to be clear, otherwise they may misunderstand, thinking we want to grab their business or something”. – CP11*  *“I feel like pharmacists are quite well equipped to deal with these things anyway. It’s things we deal with on a daily basis. I always talk to people who come in and look a little bit down and I’ll ask them, well what's going on? Come talk to me”. – CP10*  *“Often, we do have that conversation with people. Someone coming to pick you up? Someone bringing food to you? Who's cooking for you? That's the sort of thing that really helps people. If that can be formalised. Almost like a checklist if you like, when someone's in that state. Where are they at.” – CP5*  *“Those two points that you mentioned. They're not heavy topics that are outside of the pharmacist scope of practice. I think that fits within the lifestyle scope. The lifestyle advice that we can provide so that sounds really good.*  *I think that would be very good. I think we would be more than capable of being able to do that”. – CP2*  *“I think if we were to provide this whole service, we do probably [need] some sort of counselling guide. So, we probably want to get a little bit more exposed to these type of counselling activity ourselves that we can attend like maybe seminars or workshops. So, I think that's probably something that needs to be arranged. So just give the course we need to actually deliver the service”. – CP8*  *“I find most the type of patients that are more likely to need mental health intervention or someone to talk to are the ones that are suffering from chronic pain”. – CP1*  *“I don't know if chronic pain fits into it. It does really, being a long-term condition, people are particularly prone to depression and anxiety. Chronic pain - it's just a whole another story. So, there's a lot of support we often need to provide to those people, mainly around interactions”. – CP4* |

**More Quotes for Theme 3: Barriers and facilitators to implementation**

| Subthemes | Quotes |
| --- | --- |
| **Perceived accessibility and approachability of community pharmacists**        **Resource constraints**                                          **Financial and non-financial incentives**                  **Confidence and empathy**            **Training in service delivery**            **Perspectives on patient engagement with pharmacy services** | *“I think the main advantage is easy access. Obviously, pharmacy is always there. Generally, [you] don’t need to book to see the pharmacist which is really different to GP whereas you have to book some time”. – CP8*  *“Pharmacy is a safe space for them. They might just come in. Just spend a bit of time in the shop around people that they know and that we’re happy to see them. We encourage that if they ever do need somebody to talk to that they can just come down and spend a bit of time in the shop and hang out”. – CP3*  *“The barrier is definitely time and manpower to do it. So, I think, just like everything at the moment, we're just struggling to find staff. I would like to do more vaccinations. I can't really find staff to do it” – CP5*  *“I think for some pharmacies, the counselling room may not be secure/may not be soundproof or maybe just positioned in a way that doesn't feel/makes people feel that's not private enough”. – CP1*  *“With the techs, they can actually help you with the initial screening. They can work for you, but with the actual counselling and behaviour intervention, I believe the pharmacists should be doing that so I think one extra person should be sufficient”. – CP8*  *“If it allows other pharmacy staff to provide this service. If they have done some sort of form of training, it’s great because you are utilising other staff and because it does not require pharmacists to provide this intervention, in my opinion, so then you can really utilise other people's skills”. – CP1*  *“Good system in place where we can do things quite quickly. Not a lot of manual work. Maybe like electronic system, including record keeping. If it can be all electronic and done quite quickly”. – CP10*  *“The other thing is that, again I’ll go back to the integration, it needs to be integrated with what our other health team members are doing in primary care. If it helps with the connection at a system level, that would be important for me. Otherwise, I just wouldn't bother”. – CP4*  *“The second thing will be remuneration. That's important too because without that, it’d be difficult for us to provide the resources to provide it”. – CP5*  *“Big chains don’t seem to be doing like the antivirals or any of that sort of stuff. They're just focusing on prescriptions and over the counter. So the ways that a lot of more independent pharmacies are staying afloat is doing those extra services and building good relationships with their customer base through those more customer facing services”. – CP3*  *“From my point of view, pharmacy in the end is business, unfortunately. The owner is obviously there to make money and to make sure that they can actually pay for all the staff as well”. – CP8*  *“Talking about feelings in life and someone's emotion and talking about mental health is not their forte and so they may shy away from it, and I think a lot of pharmacists will need further training to be able to do that”. – CP1*  *“I think that there are some members of the team that don't have those core empathy and compassion skills. So, I just feel like there would be some members of the team that would embrace it and some members that wouldn’t”. – CP4*  *“I think [the last one] would be adequate training provided and resources for the pharmacist to feel like they could do it in a manner that's safe for those patients”. – CP3*  *“Just to mitigate any potential risks like any situations that we may not know how to handle just because we haven't had training”. – CP10*  *“Perhaps, it may even give it credibility as well for some people's minds”. – CP9*  *“Patient cooperation and interest would be another major thing. I think it's a great idea, but I have no idea if people coming off from the street would want that to be how they access mental health care. I don't know how people would feel about it”. – CP3*  *“Mental well-being service is actually probably a good phraseology because it's not confronting”. – CP6*  *“De-stigmatisation will be something that I think is quite important because obviously mental health still has a really big stigma around it”. – CP8*  *“It depends on the person too. Not everyone can do this job. I think sometimes this sort of conversation does rely a lot on personal relationship. I can't just have a locum come in and talk to this person who's upset. I don't think it's going to work very well. If it's a regular person that they have a close relationship with, then that will probably work a bit better”. – CP5* |

**More Quotes for Theme 4: Design and implementation of service**

| Subthemes | Quotes |
| --- | --- |
| **Promotion and advertisement**      **Patient identification and screening**                                **Intervention options**                      **Mode of delivery, intensity, and duration of the intervention** | *“If you want to go all out, you can have radio ads and having that advertisement online. I'm sure all the tech experts can do that”. – CP1*  *“So national type advertising, through usual media kind of means. So, TV, radio, online profiles and things”. – CP2*  *“When you know someone's having a tough time, then you can approach them and say, hey, we do actually have a service”. – CP2*  *“I think I probably prefer to talk to them, depending on the patient themselves. Some people are maybe more shy, then ideally, I'll probably prepare a questionnaire. So that's easy. So, they can do it at their own time. They can take it away with them. Fill it at their own time”. – CP8*  *“Maybe I'll like to give them some time so they can go home and have a think as well. That way it's not everything in one go. Give them some time to process everything that way. We do tell them what events that we found out and obviously they had some time to think about themselves. Maybe that way, they will be more aware of what might be going on. So, it won't be such a big shock to them. – CP8*  *“Even if you put it to the side and just did some other things for a while and then called the patient. The very least comes across the patient is that you've given it some time and thought”. – CP3*  *“So, I guess it's around education as to picking up those signs. So, change in partner, grief and loss, change in medication, post-hospitalisation, post-acute event”. – CP4*  *“The ones that are actually showing quite dangerous signs, like there is some signs of clinical depression, we would want, I assume, to refer back to the GP would be my gut feel there”. – CP7*  *“The low severity is probably just giving them the tools. Giving them like website referral, local counsellor referral, phone numbers that they can call and just giving them a life checklist. Making sure they’ve got people they can call. Making sure people are checking in on them”. – CP5*  *“Maybe it's at the simpler end of the cognitive behavioural therapy. What's the simplest approaches they use to get people on the right pathway.” – CP9*  *“I think it would need to be something similar to motivational interviewing. So something conversational and with maybe some resources”. – CP4*  *“If we get a bit of a training on brief intervention, yes, certainly something we can give definitely do. I'm 100% certain there's a training like that.*  *So, some sort of brief intervention”. – CP5*  *“I think perhaps the service can be delivered in a way that can be more flexible for pharmacies. So maybe a call back or I think nowadays, because we all have computers, we could potentially even do video call for some patients if they really want to if they couldn't come in, but phone calls usually work fine”. – CP1*  *“I think the easiest way and I feel the most effective way is in person because a lot of people actually want that personal touch in the sense that they actually want to feel close to somebody else. The best way to do that is in person”. – CP1*  *“It has to be user-friendly. So, it has to be an easy-to-follow process rather than a complicated process”. – CP7* |
